# Antioxidant Activity, and Volatile and Phytosterol Contents of *Strobilanthes crispus* Dehydrated Using Conventional and Vacuum Microwave Drying Methods

**DOI:** 10.3390/molecules24071397

**Published:** 2019-04-09

**Authors:** Lisa Yen Wen Chua, Bee Lin Chua, Adam Figiel, Chien Hwa Chong, Aneta Wojdyło, Antoni Szumny, Thomas Shean Yaw Choong

**Affiliations:** 1 School of Engineering, Taylor’s University, Lakeside Campus, No. 1, Jalan Taylor’s, Subang Jaya, Selangor 47500, Malaysia; lisacyw92@gmail.com; 2 Institute of Agricultural Engineering, Wrocław University of Environmental and Life Sciences, 37a Chełmońskiego Street, 51-630 Wrocław, Poland; adam.figiel@upwr.edu.pl; 3 School of Engineering and Physical Sciences, Heriot-Watt University Malaysia, No. 1 Jalan Venna P5/2 Precinct 5, Putrajaya 62200, Malaysia; chien_hwa.chong@hw.ac.uk; 4 Department of Fruit, Vegetable and Plant Nutraceuticals Technology, Wrocław University of Environmental and Life Sciences, 37 Chełmońskiego Street, 51-630 Wrocław, Poland; aneta.wojdylo@upwr.edu.pl; 5 Department of Chemistry, Wrocław University of Environmental and Life Sciences, Norwida 25, 53-375 Wrocław, Poland; antoni.szumny@upwr.edu.pl; 6 Department of Chemical and Environmental Engineering, University Putra Malaysia, UPM Serdang, Selangor 43400, Malaysia

**Keywords:** *Strobilanthes crispus*, drying technology, vacuum microwave drying, antioxidant activity, essential oil volatile compound, phytosterol

## Abstract

The preservation of active constituents in fresh herbs is affected by drying methods. An effective drying method for *Strobilanthes crispus* which is increasingly marketed as an important herbal tea remains to be reported. This study evaluated the effects of conventional and new drying technologies, namely vacuum microwave drying methods, on the antioxidant activity and yield of essential oil volatiles and phytosterols. These drying methods included convective drying (CD) at 40 °C, 50 °C, and 60 °C; vacuum microwave drying (VMD) at 6, 9, and 12 W/g; convective pre-drying and vacuum microwave finish drying (CPD-VMFD) at 50 °C and 9 W/g; and freeze-drying (FD). GC–MS revealed 33 volatiles, and 2-hexen-1-ol, 2-hexenal, 1-octen-3-ol, linalool, and benzaldehyde were major constituents. The compounds β-sitosterol and α-linolenic acid were the most abundant phytosterol and fatty acid, respectively, in fresh *S. crispus*. The highest phenolic content was achieved with CD at 60 °C. The highest antioxidant activity was obtained with CD at 40 °C and VMD at 9 W/g. On the contrary, the highest total volatiles and phytosterols were detected with CD at 50 °C and VMD at 9 W/g, respectively. This study showed that CD and VMD were effective in producing highly bioactive *S. crispus*. A suitable drying parameter level, irrespective of the drying method used, was an important influencing factor.

## 1. Introduction

*Strobilanthes crispus*, known as “pecah beling” or “Hei Mian Jiang Jun”, is native to various countries, such as Malaysia and Indonesia. Its leaves are used as traditional medicine in the form of infused fresh or dried leaves. The intake of its herbal preparation became popular as an alternative treatment to prevent diseases and increase overall well-being. Al-Henhena et al. (2015) showed that *S. crispus* extract is high in antioxidant activity because of various phenolic constituents, such as caffeic acid, ferulic acid, kaempferol, and luteolin [1,2,3].

Drying is an effective preservation step that involves reducing the moisture content of a freshly harvested material, thereby extending a product’s shelf life. Conventional drying methods, such as convective drying (CD) and freeze-drying (FD), are characterized by low drying rates, especially in the falling rate period [4]. New drying technologies, such as vacuum microwave drying (VMD) and convective pre-drying followed by vacuum microwave finish drying (CPD-VMFD), can address the limitations of conventional drying. Both drying methods generated products of high bioactive retention, essential oil content, and antioxidant activity in the drying of basil [5], oregano [6], thyme [7], and beetroot [8]. VMD is a hybrid drying technology that combines microwave and vacuum drying. During VMD, microwaves interact with water molecules in the whole volume of a product, leading to volumetric heating. A large vapor pressure formed at the central region of a material facilitates quick moisture removal [8,9].

In VMD combined with CD, a material is pre-dried with CD and completely dried through VMD. This combined method, known as CPD-VMFD, maximizes the benefits of both drying methods. VMFD is usually applied at the end stage of drying to remove bound water from a product during the falling rate period, thereby effectively reducing drying time [9].

This study aimed to evaluate the effects of different drying methods, namely conventional and new drying methods, on the drying kinetics, phenolic content, volatile and phytosterol content, antioxidant activity, water activity (a_w_), and color of dried *S. crispus*. The fatty-acid profile was also determined to verify the active constituents in *S. crispus*. VMD methods are yet to be utilized to dry *S. crispus*. As such, this study also aimed to confirm the potential of this new innovative technology. Determining good drying applications is important for manufacturing new dried forms of *S. crispus* with high bioactivity. The drying kinetics were modeled with thin-layer models to integrate experimentally obtained data into industrial applications. Drying is also one of the most energy-intensive operations in the food industry. This study also considered the relationship of drying kinetics with the energy input of a drying operation and used specific energy consumptions to provide an indication of the cost effectiveness of *S. crispus* drying which is yet to be reported.

## 2. Results

### 2.1. Drying Kinetics

The drying kinetics of *S. crispus* leaves dried using CD, VMD, and CPD-VMFD were best described by the modified Page model (Equation (1)) based on the highest *R^2^* and the lowest root-mean-square error (RMSE).

(1) $$MR=a\cdot \exp\;\left(-k\cdot t^{n}\right),$$

where *a* is the model constant, *k* is the drying constant, and *n* is the dimensionless empirical constant.

**Figure 1:**
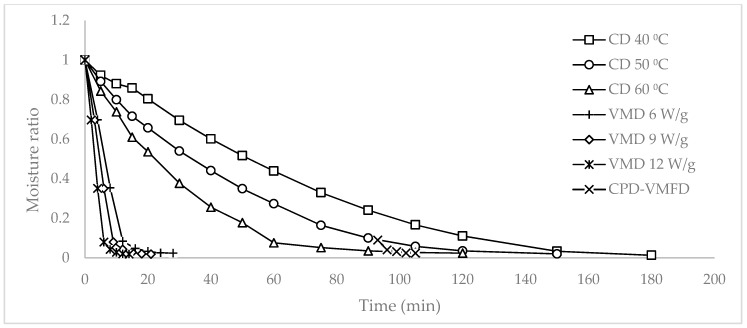
The relationship between moisture ratio and time for the drying of *Strobilanthes crispus* with convective drying (CD) at 40, 50, and 60 °C, vacuum microwave drying (VMD) at 6, 9, and 12 W/g, and convective pre-drying followed by vacuum microwave finish drying (CPD-VMFD) at 50 °C and 9 W/g.

Figure 1 shows the relationship between the moisture ratio (MR) and the time consumed for the leaves to dry under various drying methods and conditions. CD at 40 °C and 50 °C required 180 and 150 min to complete drying, respectively. By contrast, CD at 60 °C required 120 min. VMD at 6, 9, and 12 W/g required shorter drying durations of 28, 21, and 14 min, respectively. The lengthy drying process of CD was shortened to 105 min by introducing VMFD as observed in CPD-VMFD.

**Figure 2:**
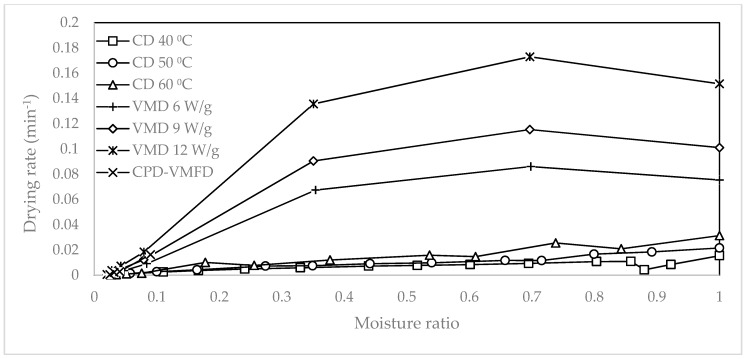
The relationship between drying rate and moisture ratio for the drying of *S. crispus* with CD at 40, 50, and 60 °C, VMD at 6, 9, and 12 W/g, and CPD-VMFD at 50 °C and 9 W/g.

Figure 2 illustrates the relationship between the drying rate and the MR. The drying rates between CD and VMD largely differed. VMFD increased the drying rate to 0.016 min^−1^ from a low drying rate of 0.003 min^−1^ during CPD at 90 min. When the moisture content of the leaves was high during the initial drying period, the drying rate increased with time regardless of the drying methods. In CD, surface water evaporated easily to the surrounding air through external diffusion. The removal of surface water produced a moisture gradient in the material, and the removal of moisture at this stage depended on internal diffusion that occurred at a slow rate. In VMD and VMFD, the high drying rate observed at the beginning could be explained by the high amount of available water molecules, resulting in high microwave energy absorption. However, the amount of moisture that reduced as drying progressed corresponded to the low absorption of microwave energy, resulting in the decreasing drying rate.

The maximum drying rates observed for VMD at 6, 9, and 12 W/g were 0.086, 0.115, and 0.173 min^−1^, respectively. In CD at 40 °C, 50 °C, and 60 °C, the maximum drying rates were 0.015, 0.022, and 0.031 min^−1^, respectively. In VMD, the drying duration of *S*. *crispus* leaves was reduced from 81% to 92%. When VMFD at 9 W/g was introduced to CPD at 50 °C, the drying rate increased from 0.003 min^−1^ to 0.044 min^−1^. This condition could be attributed to the volumetric heating of VMD in which high internal moisture could be effectively removed at a fast rate, thereby shortening the drying time from 150 min to 105 min.

Increasing the parameter level, that is, microwave power and hot air temperature, led to an increase in the drying rates. As the microwave power increased from 6 W/g to 9 W/g, the average drying rate also increased from 0.035 min^−1^ to 0.047 min^−1^. On the contrary, when the microwave power increased from 9 W/g to 12 W/g, the average drying rate increased from 0.047 min^−1^ to 0.070 min^−1^. When hot air temperature increased by 10 °C, a marginal improvement was noted. The average drying rate also increased from 0.008 min^−1^ to 0.009 min^−1^ by increasing the hot air temperature from 40 °C to 50 °C. Similarly, the average drying rate increased from 0.009 min^−1^ to 0.010 min^−1^ when temperature increased from 50 °C to 60 °C.

### 2.2. Antioxidant Activity

The analysis of the antioxidant results revealed that CD showed potential for preserving antioxidant constituents in *S. crispus* despite the long dying duration of 150–180 min. Samples dried using CD at 40 °C (2,2′-azinobis(3-ethylbenzthiazoline-6-sulfonic acid) (ABTS): 5.08, and ferric-reducing antioxidant power (FRAP): 5.98 µM Trolox/100 g dry weight (dw)), and CD at 60 °C (ABTS: 4.67, and FRAP: 5.67 µM Trolox/100 g dw) achieved the highest antioxidant values that were significantly higher than those of FD (ABTS: 3.57, and FRAP: 4.48 µM Trolox/100 g dw). The long exposure to heat and oxygen did not reduce the antioxidant activity. In previous studies, antioxidant activities and polyphenol contents in heat-treated peppermint, oregano, *Artemisia annua*, and various Lamiaceae herbs, such as rosemary, marjoram, oregano, sage, thyme, and basil [10,11], increased, indicating that phenolic compounds at a partial state of oxidation are exposed to oxygen and may show a high antioxidant activity.

CPD-VMFD (50 °C, 9 W/g) and CD at 50 °C produced the lowest ABTS, FRAP, and total phenolic content (TPC). A good agreement between these values is shown in Table 1. We assumed that degradative enzymes in *S. cripus* could achieve optimum activity at 50 °C. Therefore, phenolic and antioxidant compounds were likely degraded. Both drying methods which operated at the same temperature of 50 °C consistently yielded samples with low antioxidant activities and TPC values.

It was observed that the FRAP values of fresh samples were lower than those of some dried samples. Thermal treatment in drying may have caused an easier release of cell constituents from plant cells [12]. This is because heat is known to exert modifications to the microstructure of plant cells, thereby reducing plant cell integrity [13]. This allows for the easy exit of antioxidants from plant cells to the extraction solvent. This is in agreement with past studies which reported that dried leaves have the tendency to show increased bioactivity [14,15]. Furthermore, loss of moisture that occurs in leaves during drying, which is categorized as abiotic stress, may have caused the formation and accumulation of phenolic compounds, thereby increasing the overall antioxidant activity [16]. Additionally, the increase in antioxidant activity in dried leaves compared to fresh leaves may be the result of Maillard reaction, producing Maillard reaction products (MRPs), with antioxidant power [17].

**Figure 3:**
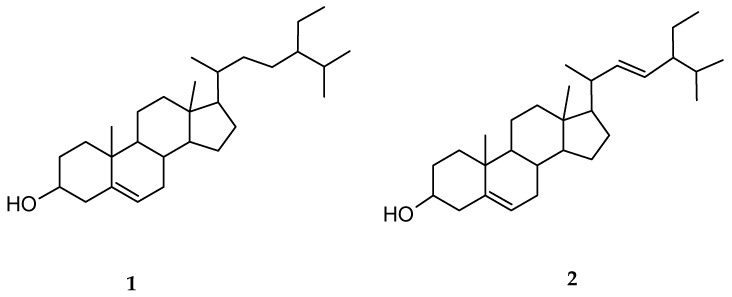
Chemical structures of β-sitosterol (**1**) and stigmasterol (**2**).

Table 1 also shows the TPC of fresh and dried *S. crispus*. The TPC value obtained from the fresh sample was 1222 mg/100 g dw, comparable with the TPC value of 1262 mg/100 g dw described in a previous study [2]. All of the drying methods led to considerable losses of phenolic compounds, including FD that involved no heat treatment. The low TPC of the heat-treated samples could be caused by the thermal and oxidative degradation of phenolic compounds. In VMD, TPC considerably decreased when microwave power was 12 W/g. In CD, a higher phenolic concentration was retained at 40 °C and 60 °C than at 50 °C. The highest TPC (1086.71 mg/100 g dw) was detected with CD at 60 °C among the drying methods. Overall, the TPC and antioxidant values did not correlate well. Therefore, this suggests that it is possible that other antioxidant compounds other than phenolic compounds, such as essential oil volatile components, were present in *S. crispus*, which contributed to the overall antioxidant activity. Antioxidant compounds derived from plants such as phenolic compounds and terpene derivatives are widely reported to exhibit antioxidative properties. In our study, the most abundant phytosterols identified in *S. crispus* were β-sitosterol and stigmasterol, both of which were reported to have an antioxidant effect [18]. Phytosterols are triterpenes and are characterized by the tetracyclic cyclopenta (α) phenanthrene structure [19]. The chemical structures of both compounds are shown in Figure 3.

**Figure 4:**
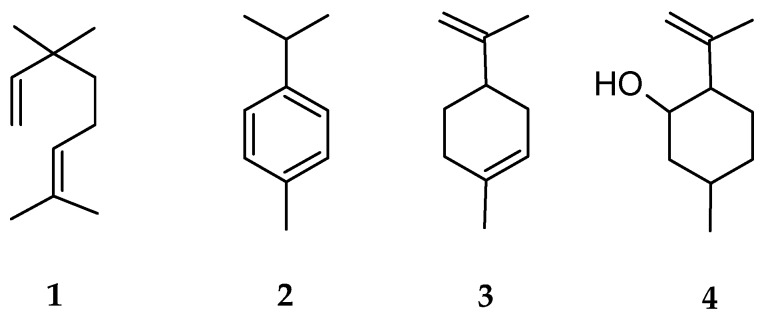
Chemical structures of linalool (**1**), *p*-cymene (**2**), limonene (**3**), and isopulegol (**4**).

The volatile composition of *S. crispus* was also investigated in this study; 2-hexen-1-ol 2-hexenal 1-octen-3-ol linalool, and benzaldehyde constituted the major compounds of *S. crispus* volatile content. However, the antioxidant activity of *S. crispus* is more likely to be contributed by terpenoids present in *S. crispus*, such as linalool, *p*-cymene, limonene, and isopulegol. Figure 4 shows the chemical structures of these compounds. Linalool, which is the most dominant terpenoid in *S. crispus*, is a monoterpene alcohol reported to have an antioxidant effect [20]. A vast number of terpenoids are reported as potential antioxidant molecules due to their ability to interact with free radicals. The most effective antioxidant compounds are the ones that disrupt the free-radical chain reaction. These antioxidants usually consist of aromatic or phenolic rings with the ability to donate H^+^ to free radicals produced during oxidation, becoming radicals themselves. The resonance delocalization of electrons in the aromatic ring will act to stabilize these radical intermediates [21]. Therefore, the H^+^-donating abilities are predicted to contribute to the structure–activity relationship [22]. With the presence of various constituents in the extract of *S. crispus*, such as terpenoids and phytosterols, the overall antioxidant activity may have been contributed by the synergistic interactions of these compounds. Synergism between antioxidants involves several mechanisms. Synergism occurs when there is a combination of two or more antioxidants with different antioxidant mechanisms, or when there is a combination of two or more different free-radical scavengers, in which an antioxidant is found to be regenerated by other antioxidants. More specifically, an antioxidant is characterized as a sacrificial antioxidant which is oxidized to exert protection on another antioxidant. Furthermore, regeneration of a primary antioxidant, with higher reduction potential by a secondary antioxidant (co-antioxidant), with less reducing power, is able to contribute to a higher net interactive antioxidant effect compared to the total individual antioxidant effects [23]. Synergistic interactions between antioxidant compounds derived from hops extract were reported in a previous study [24].

Drying intensity slightly influenced the antioxidant activity and TPC. However, CD at a low temperature of 40 °C produced a sample with the highest antioxidant activity among the convective-dried samples (50 °C and 60 °C). The antioxidant activity obtained with a moderate VMD treatment intensity of 9 W/g was relatively better than that found with VMD at 6 and 12 W/g.

### 2.3. Volatile Compounds in Fresh and Dried S. crispus

**Figure 5:**
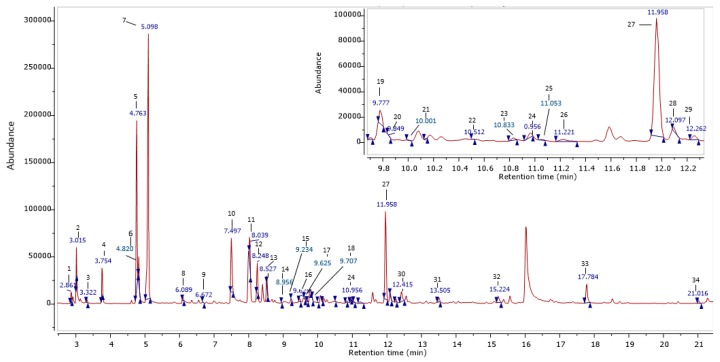
GC–MS chromatogram of fresh *S. crispus* (for peak identification, see Table 2).

Table 2 shows the volatile compounds of fresh and dried *S. crispus* leaves extracted using headspace solid-phase microextraction (HS-SPME). A typical chromatogram showing the volatile compounds of *S. crispus* is shown in Figure 5. The concentration of the total volatiles in the fresh sample was 361.23 mg 100 g^−1^ db (dry basis). The most abundant volatiles in fresh *S. crispus* were 2-hexen-1-ol (84.11 mg 100 g^−1^ db), 2-hexenal (51.18 mg 100 g^−1^ db), 1-octen-3-ol (36.10 mg 100 g^−1^ db), linalool (34.91 mg 100 g^−1^ db), and benzaldehyde (27.61 mg 100 g^−1^ db). The volatiles present in *S. crispus* could be categorized under the following chemical groups: alcohol (50.2%), aldehyde (24.3%), monoterpenoid (10.9%), ketone (4.1%), ester (4.0%), pyridine (3.4%), monoterpene (1.4%), and enone (0.9%). Other compounds were also found, such as cycloalkenes (0.5%), cyclohexenones (0.3%), and unidentified volatiles, represented 0.1% of the total volatiles.

All of the drying methods resulted in considerable losses in volatile content (Table 2). The volatile content obtained with CD at 50 °C and 60 °C was higher than that produced by other drying methods. This finding showed that CD at 50 °C was suitable for the drying of *S. crispus* in terms of retaining volatiles. However, we assumed that VMD and CPD-VMFD would retain a higher concentration of volatiles because the drying rate could be increased by the volumetric heating of microwaves. Microwave treatment could also cause a large disruption of the orderly tissue structure to improve mass and heat transfer, thereby increasing the drying rate. However, in this study, the increasing microwave power of VMD led to high losses in volatile content possibly caused by the high intensity of the drying treatment. Similar results were reported in the drying of basil [5], marjoram [6], rosemary [25], and thyme [7].

Although CD had few disadvantages, such as a long drying time and being prone to volatile oxidation, convective-dried leaves are known to develop a partially dried layer on the surface following CD [6,26]. This crust layer is important because it forms a barrier that limits losses in volatiles [27]. Buchaillot et al. (2009) reported that a temperature of 50 °C was advantageous to forming this layer, whereas low temperatures of 30 °C and 40 °C resulted in a high volatile loss in lemon myrtle leaves [27]. This finding agreed with our results, that is, a high amount of volatiles were retained through CD at 50 °C (17.4 mg 100 g^−1^ db). At a high temperature of 60 °C, the concentration of volatiles was reduced by 13.8%.

Our results also agreed with a previous study which reported that the VMD of rosemary leads to higher losses in volatile content (61.9 g kg^−1^ dw) than CD (87.2 g kg^−1^ dw) [25]. It is postulated that VMD is associated with higher losses of volatiles as the enhanced internal vapor generated in plant tissues causes an increased porosity of cellular structure compared to convective-dried leaves [28,29]. The porous cellular structure in turn facilitates the escape of a higher amount of volatiles to the surroundings. However, it was reported that the VMD of sweet basil retains higher amounts of volatiles than CD does [5]. Therefore, no single drying method is consistently effective in ensuring high volatile retention in herbs, and losses in volatile content may vary considerably from one species of herb to another [30]. Drying inherently results in the loss of volatiles, as moisture evaporated during drying acts as a carrier to which volatiles dissolve and escape to the surroundings [6,31]. Higher losses were reported to occur during the initial drying period, as a higher amount of moisture is evaporated; therefore, it is crucial to determine the relationship between amount of water evaporated and volatile content. Drying should be stopped at a moisture content that corresponds to a safe microbial stage to minimize unnecessary volatile loss [30].

Since water acts as a solvent in which volatiles are dissolved; when a high amount of water is removed from the sample, a high volatile content could be lost. Therefore, the low total volatile concentration of the freeze-dried samples could be attributed to the high removal of water during FD, considering that the freeze-dried samples had the lowest a_w_ of 0.0245. Moreover, the porous structure of freeze-dried leaves may have facilitated the diffusion and escape of volatiles [26,27]. The retention level of volatiles depends on an individual compound’s volatility and its affinity toward water [31,32]. The hydrophobicity of a particular volatile compound is advantageous to limiting losses in volatiles [6].

### 2.4. Phytosterol Analysis

GC–MS analysis identified nine phytosterols, and the respective concentrations influenced by the different drying methods and drying intensities are shown in Table 3. The major phytosterols in fresh *S. crispus* were β-sitosterol, stigmasterol, and campesterol, with concentrations of 1476.67, 1207.96, and 791.57 mg 100 g^−1^ db, respectively. Considerable losses of phytosterol were noted in dried samples. Rudzinska et al. (2009) reported that the stability of phytosterols is affected by chemical structure, processing temperature, and time [33]. In our study, VMD at 9 W/g retained the highest phytosterol content (542.48 mg 100 g^−1^ db) because of the favorable drying condition of the reduced oxygen and the accelerated drying process. The freeze-dried samples had the lowest phytosterol content (462.07 mg 100 g^−1^ db). Overall, moderate hot air temperature (CD at 50 °C) and microwave power (9 W/g) retained higher phytosterol contents. Soupas et al. (2004) reported that several factors, such as temperature and heating duration, phytosterol structure, and lipid matrix composition, affect the oxidative stability of phytosterols [34]. Gawrysiak-Witulska et al. (2015) demonstrated that high phytosterol degradation in rapeseed is related to high drying temperature [35]. With respect to the results of our study, the applied moderate drying intensity effectively reduced the drying duration to prevent the high extent of degradation and oxidation due to long drying time. However, such intensity induced a heat degradative effect on phytosterols. In addition, phytosterols are most likely lost through an oxidation process, especially during CD. Similar findings were reported by Rudzinska et al. (2009) [33]. With the application of appropriate hot air temperature and microwave power, attaining relatively higher retentions of phytosterols was possible.

Barriuso et al. (2012) indicated that the degradation level of phytosterols depends on their chemical structure [36] and investigated the degradation behavior of β-sitosterol, stigmasterol, and campesterol at 180 °C. They found that campesterol is highly susceptible to degradation, similar to β-sitosterol, and with stigmasterol less susceptible. However, in our study, the highest percentage reduction of phytosterols among the fresh samples and the samples subjected to VMD at 9 W/g was observed in campesterol (91.6%), followed by stigmasterol (87.8%) and β-sitosterol (84.5%).

### 2.5. Fatty-Acid Profile

**Figure 6:**
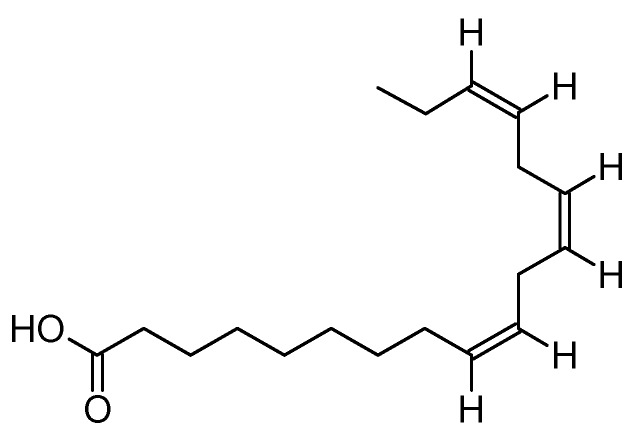
Chemical structure of α-linolenic acid.

The fatty-acid profile of *S. crispus* is shown in Table 4. The major fatty acid was α-linolenic acid, followed by linoleic acid and palmitic acid, constituting 58%, 12.49%, and 10.48% of the total fatty acids present, respectively. α-Linolenic acid is an 18-carbon, polyunsaturated fatty acid (Figure 6) and is a widely known antioxidant that may have contributed to the overall antioxidant activity of *S. crispus*. α-Linolenic acid is essential for overall well-being, as the human body does not have the required enzyme for the synthesis of this compound, which must be acquired from dietary sources [37].

### 2.6. Specific Energy Consumption

**Figure 7:**
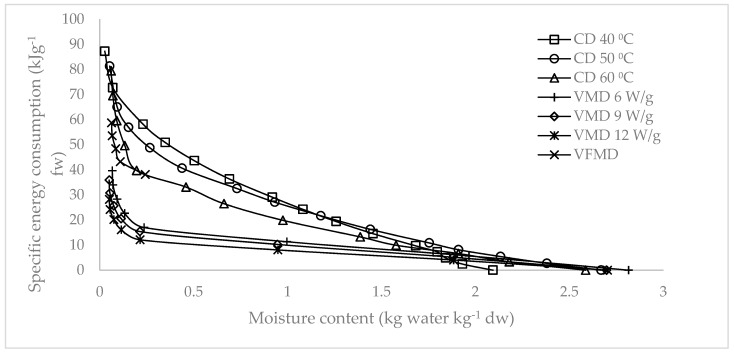
Specific energy consumption of *S. crispus* leaves per gram of fresh weight dried using CD, VMD, and CPD-VMFD.

**Figure 8:**
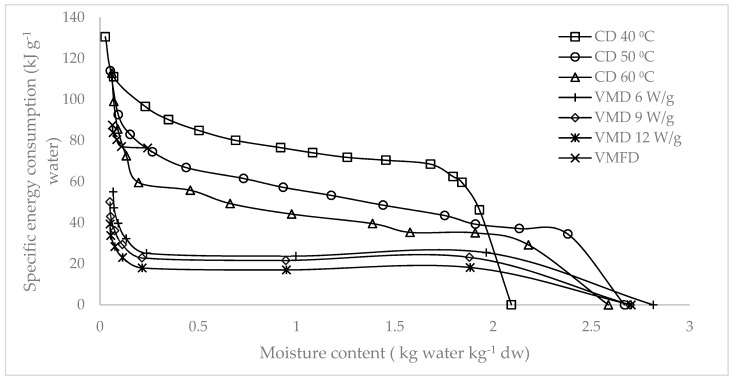
Specific energy consumption of *S. crispus* leaves per gram of water evaporated using CD, VMD, and CPD-VMFD.

**Figure 9:**
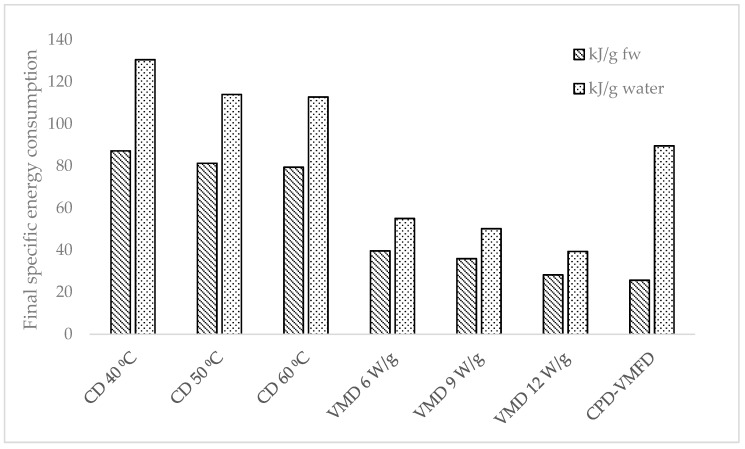
Final specific energy consumptions of CD, VMD, and CPD-VMFD of *S. crispus* leaves.

Figure 7 and Figure 8 illustrate the specific energy consumption expressed in (1) kilojoules per gram of fresh weight and (2) kilojoules required for the removal of 1 g of water. Specific energy consumptions increased as the moisture content decreased. A steep increase was observed at the final drying stage. Figure 9 shows the final specific energy consumptions of CD, VMD, and CPD-VMFD. CD consumed more energy than VMD did. However, the high final specific energy of CD at 50 °C could be reduced with the application of VMFD, reducing specific energy consumptions from 81.28 kJ/g fresh weight (fw) to 58.70 kJ/g fw and from 113.99 kJ/g water to 87.59 kJ/g water. The final specific energy consumption also reduced as hot air temperature and microwave power increased in CD and VMD, respectively. High drying intensities corresponded to a short drying duration, thereby reducing the specific energy consumption. The specific energy consumption can be considered as the heat energy input which affects the chemical changes in the raw material during drying for a certain time at different drying parameters. The highest energy input distributed over a long time period at the lowest temperature (40 °C) was beneficial for antioxidant activity and TPC (Table 1). On the other hand, decreasing the energy input and drying time by increasing temperature to 50 and 60 °C increased the content of phytosterols (Table 3). This enhancing effect consisting of decreasing the energy input and drying time was confirmed by the highest content of phytosterols achieved for VMD at 9 W/g. Decreasing the energy input and drying time was also favorable for the concentration of volatile compounds in the case of CD samples (Table 2).

### 2.7. Water Activity Analysis

Water activity (a_w_) is a measure of the degree of water bound to a food product, and it is a key parameter that influences not only microbial systems, but also the chemical and physical stability of food products. The measured a_w_ of fresh and dried leaves is shown in Table 5. The a_w_ of the leaves dried using the drying treatments ranged from 0.0245 to 0.1577. The FD samples showed the lowest a_w_. The a_w_ of the samples subjected to CD at 50 °C and 60 °C and CPD-VMFD was lower than that of the samples subjected to CD at 40 °C and VMD (6 and 12 W/g). A high applied temperature coupled with a long drying duration in CD ensured the low residual moisture in the leaves. Increasing temperature in CD resulted in a low a_w_. Applying different levels of microwave power exhibited no evident trends. Dried products should have a low a_w_, ranging from 0.60 to 0.80, because spoilage bacteria and most molds are inhibited below a_w_ of 0.91 and 0.80, respectively [38]. Therefore, the low a_w_ of the dried leaf samples shows that all of the drying treatments were effective in ensuring microbiologically safe products. The same conclusion concerns the chemical and physical stability of the dried product. The a_w_ is a good indicator of biochemical degradation reactions that may take place during storage. For instance, it is known that Maillard reaction occurs at an optimum a_w_ of 0.65. Therefore, the determination of a_w_ is not only crucial in the prediction of shelf-life, but is also informative in terms of understanding biochemical reactions that may take place in relation to the a_w_ value [39].

### 2.8. Color Analysis

**Figure 10:**
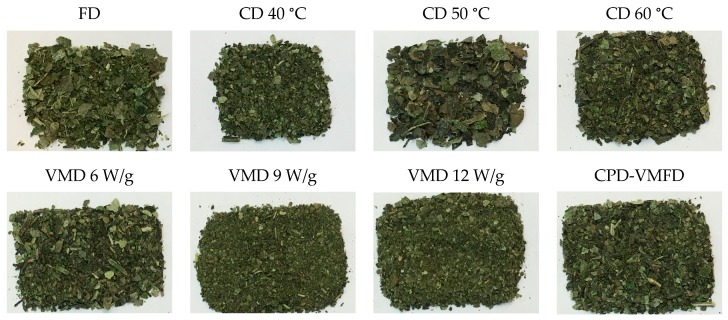
Image of ground *S. crispus* dried using FD, CD at 40, 50, and 60 °C, VMD at 6, 9, and 12 W/g, and CPD-VMFD at 50 °C and 9 W/g.

Color is often perceived as an index of freshness and is associated with a nutritional value. Therefore, color is an important cue used by consumers to evaluate the quality of a certain product. Among factors contributing to the changes in the color profile of dried herbs are hydrolysis, caramelization, and nonenzymatic and enzymatic reactions. Chlorophylls are unstable and may undergo transformation or degradation, forming olive-brownish and greenish derivatives or colorless substances. Figure 10 shows the image of dehydrated *S. crispus* dried using various drying methods. The visual images were confirmed by the results obtained from instrumental measurement of color (Table 6). In our study, the freeze-dried samples had the lightest (highest L* value) and greenest (lowest a* value) color. The convective-dried samples at 40 °C and the VMD-treated samples (6, 9, and 12 W/g) also exhibited lightness and greenness values comparable with those of the freeze-dried samples. The freeze-dried samples effectively preserved the color of herbs because heat was not involved in dehydration, in addition to a reduced oxygen condition. This finding was possible because the most widely occurring process in plants is the conversion of chlorophyll *a* and *b* to pheophytins (olive green) during heat treatment. Drying inherently led to the mechanical disruption of the cytoplasmic membrane, thereby allowing membrane permeability to increase, causing cellular acids to be released and degrading chlorophylls to pheophytins [40]. VMD conditions (reduced oxygen, low boiling temperature with reduced pressure, and short drying durations) effectively preserved the color of herbs. High hot air temperatures (50 °C and 60 °C) in the convective-dried and convective pre-dried samples produced dark leaves because the subjection to high drying temperature for a long duration might have intensified chlorophyll degradation.

In b*, a high b* (yellow) was obtained for the freeze-dried samples, indicating that carotenoids were preserved well during FD, followed by VMD at 12, 6, and 9 W/g, and all of these processes exposed the samples to a reduced oxygen condition. Carotenoids are susceptible to oxidation. As such, this result was expected. Carotenoids were preserved to a greater extent with CPD-VMFD at low hot air temperature (40 °C) and exposure to oxygen than with CD at 50 °C and 60 °C. For the fresh samples, the period between harvest and analysis could have allowed an extensive chlorophyll degradation to occur because of the high water content in the samples (a_w_ of 0.9879). Hence, fresh leaves appeared dark, and their green and yellow intensity decreased.

## 3. Materials and Methods

### 3.1. Chemicals and Reagents

Folin–Ciocalteu reagent, sodium carbonate, gallic acid, FRAP reagent, acetate buffer, acetic acid, 2,4,6-tripyridyl-1,3,5-triazine (TPTZ), 2,2′-azinobis(3-ethylbenzthiazoline-6-sulfonic acid) (ABTS), potassium persulfate, 6-hydroxy-2,5,7,8-tetramethylchroman-2-carboxylic acid (Trolox), *p*-methoxyphenol, chloroform/(folsch), BF_3_/MeOH, potassium hydroxide, hexane, sodium chloride, methanol, hydrochloric acid, magnesium sulfate, pyridine, and *N*,*O*-bis(trimethylsilyl)trifluoroacetamide (BSTFA) were purchased from Sigma-Aldrich (Steinheim, Germany).

### 3.2. Plant Sample Preparation and Drying

*S. cripus* leaves were purchased from TKC nursery (Seremban, Malaysia) and verified at the Forest Research Institute of Malaysia (voucher specimen: 023/18). The moisture content of the fresh samples was gravimetrically determined by drying the leaves to a constant mass through vacuum-oven drying at 60 °C for 24 h. The leaves were divided into 40 g of samples and dried using four drying protocols. Drying was stopped when the mass loss of the consecutive samples was 0.05 g or less. For VMD and VMFD, the leaf samples were periodically removed from the vacuum microwave dryer to measure the temperature using an infrared thermometer (Flir i50, Portland; OR, USA). The obtained temperature was assumed to represent the maximal temperature of the sample.

#### 3.2.1. CD

The samples were spread on trays and placed on top of the drying chambers of a convective hot air oven at 40 °C, 50 °C, and 60 °C.

#### 3.2.2. VMD

The samples were dried in a vacuum microwave dryer (Plazmatronika, Wroclaw, Poland) at 6, 9, and 12 W/g and placed in a glass container connected to a vacuum system with pressure ranging from 4 kPa to 6 kPa. The glass container was rotated at 6 rpm throughout the drying process and equipped with a fan to create an air stream with a velocity of 1 m s^−1^ and a temperature of 22 °C and, thus, prevent local overheating.

#### 3.2.3. CPD Followed by VMD

The samples were pre-dried at 50 °C in a convective hot air oven constructed in the Institute of Agriculture Engineering (Wroclaw, Poland). Drying was stopped after 90 min, and a moisture content of 0.2666 kg water kg^−1^ dw was achieved. The pre-dried leaves were then dried with VMD (Plazmatronika, Wroclaw, Poland) at 360 W [5,41].

#### 3.2.4. FD

FD was performed in a freeze dryer (OE-950, Labor MIM, Budapest, Hungary) at a pressure of 65 Pa and a temperature of −60 °C. The temperature of the heating plate reached 30 °C. The freeze-dried samples were used as control samples.

### 3.3. Drying Kinetics

The drying kinetics of *S. crispus* leaves through CD, VMD, and CPD-VMFD were based on the mass losses of samples. MR was obtained using Equation (2).

(2) $$MR=\frac{M\left(t\right)-M_{e}}{M_{0}-M_{e}},$$

where M(t) is the moisture content (kg H_2_O/kg dry matter (dm)) after drying time *t*, $M_{e}$ is the equilibrium moisture content (kg H_2_O/kg dm), and $M_{0}$ is the initial moisture content (kg H_2_O/kg dm). At the final stage of drying, the equilibrium moisture content ($M_{e}$) was obtained as an asymptotic value of the function fitted to the experimental points by using Table Curve 2D Windows v2.03 (Jandel Scientific Software, San Rafael, CA, USA). Three commonly mentioned models in the literature were used to select mathematical models that best described the drying process (Table 7).

### 3.4. Calculation of Energy Consumption

#### 3.4.1. Energy Consumption of CD and VMD Methods

The energies consumed during CD ($E_{C}$) and VMD ($E_{VM}$) were determined on the basis of Equations (3) and (4).

(3) $$E_{C}=\left(\frac{N_{f}}{6}+N_{h}\right)\times t,$$

where $N_{f}$ (kilowatts) is the power consumed by the fan supplying air to six pipes that were fixed with electric heaters with a power consumption of $N_{h}$ (kilowatts), and t is the drying duration (seconds).

(4) $$E_{VM}=\left(\frac{N_{M}}{\eta_{M}}+N_{V}+N_{e}\right)\times t,$$

where $N_{M}$ is the output power of magnetrons (kilowatt), $\eta_{M}$ is the efficiency of magnetrons, $N_{V}$ is the power consumption (kilowatts) of the vacuum pump, $N_{e}$ is the power consumption of the engine (kilowatts) that rotates the container in the dryer, and t is the drying duration (seconds).

#### 3.4.2. Specific Energy Consumption

The specific energy consumptions of CD, VMD, and CPD-VMFD were calculated on the basis of the ratio of energy consumed to the initial mass m (grams) of the leaf samples and the ratio of energy consumed to the mass of water W (grams) expelled from the leaf samples.

The specific energy consumptions of CD, represented as $E_{C_{m}}$ $\left(kJ\;g^{-1}\;fw\right)$ and $E_{C_{W}}$ $\left(kJ\;g^{-1}\;\mathrm{water}\right),$ were calculated using Equations (5) and (6), respectively.

(5) $$E_{C_{m}}=\frac{E_{C}}{m}$$

(6) $$E_{C_{W}}=\frac{E_{C}}{W}$$

Equations (7) and (8) show the specific energy consumptions of VMD, namely, $E_{VM_{m}}$ $\left(kJ\;g^{-1}\;fw\right)$ and $E_{VM_{W}}$ $\left(kJ\;g^{-1}\;\mathrm{water}\right),$ respectively.

(7) $$E_{VM_{m}}=\frac{E_{VM}}{m}$$

(8) $$E_{VM_{W}}=\frac{E_{VM}}{W}$$

Equations (9) and (10) present the value of the specific energy consumptions of CPD-VMFD, namely $E_{C-VM_{m}}$ $\left(kJ\;g^{-1}\;fw\right)$ and $E_{C-VM_{W}}$ $\left(kJ\;g^{-1}\;water\right),$ respectively. Both equations are the ratios of the sum of energy $E_{C}$ and $E_{VM}$ to the mass of the initial sample m (grams) and the ratio of the sum of energy $E_{C}$ and $E_{VM}$ to the mass of water (grams) from CPD ($W_{C}$) and VMFD ($W_{VM}$) removed from the leaf sample, respectively.

(9) $$E_{C-VM_{m}}=\frac{E_{C}+E_{VM}}{m};$$

(10) $$E_{C-VM_{m}}=\frac{E_{C}+E_{VM}}{W_{C}+W_{VM}}.$$

### 3.5. Extraction of Polyphenol Compounds

Approximately 0.3 g of ground samples was measured, placed in test tubes, and added with 80% aqueous methanol (0.7 mL) and 1% HCl. The suspension in the test tubes was stirred, sonicated twice for 15 min and left to stand at 4 °C for 24 h. The extract was centrifuged at 15,000 rpm (MPW-360R, Warsaw, Poland) for 10 min, and the resultant supernatants were collected.

### 3.6. Antioxidant Activity Analysis

#### 3.6.1. ABTS^●+^ Radical-Scavenging Assay

An ABTS radical-scavenging assay was conducted to evaluate the free-radical-scavenging activity as previously described [42]. ABTS was dissolved in water to obtain a concentration of 7 mM. ABTS stock solution was reacted with 2.45 mM potassium persulfate to form an ABTS radical cation (ABTS^●+^). This mixture was left to stand in the dark at room temperature for 12–16 h before it was used in the assay. This solution was diluted with redistilled water to obtain an absorbance of 0.700 ± 0.02 at 734 nm. For the antioxidant activity analysis, 300 µL of the diluted radical solution was mixed with 20 µL of the extracted supernatant. Absorbance was read at 734 nm after 6 min. Determinations were performed in triplicate. The obtained results were expressed relative to micromolar Trolox per 100 g of dry weight in terms of Trolox equivalent antioxidant capacity.

#### 3.6.2. FRAP Assay

FRAP assay, previously described by Benzie and Strain (1996), was used as a measure of antioxidant potential [36]. For FRAP reagent preparation, acetate buffer (300 µM, pH 3.6) was mixed with 10 µM TPTZ in 40 µM HCl and 20 µM of FeCl_3_ at a ratio of 10:1:1 (*v*/*v*/*v*). Subsequently, 300 µL of FRAP reagent was thoroughly mixed with 10 µL of the sample solution. Absorbance was read at 593 nm after 10 min. All of the prepared solutions were used on the same day of the preparation. A standard curve with different concentrations of Trolox was constructed. Determinations were performed in triplicates.

### 3.7. Total Phenolic Content

The total phenolic content was estimated by using the Folin–Ciocalteu method as described by Gao et al. (2000) [36]. A leaf extract of 0.1 mL was added to 0.2 mL of Folin–Ciocalteu reagent and 2 mL of water. This mixture was incubated at room temperature for 3 min, added with 1 mL of 20% sodium carbonate and incubated at room temperature for 1 h. Absorbance was determined using a ultraviolet–visible light (UV–Vis) spectrophotometer (Shimadzu, UV-2401 PC, Kyoto, Japan) at a wavelength of 765 nm. Quantification was based on the gallic acid standard curve, and results were expressed as gallic acid equivalence in milligrams per 100 g of dry weight. Determinations were performed in triplicate.

### 3.8. Analysis of Volatile Compounds

Volatiles were analyzed using HS-SPME method. Crushed, dried, and fresh leaf samples of approximately 0.25 g were added in glass vials with 2 µg of *p*-methoxyphenol as an internal standard. Glass vials were covered with a polytetrafluoroethylene (PTFE)/silicone septum and subsequently placed in a water bath at 60 °C for equilibration for 10 min. Extraction was performed using SPME fiber (Supelco, Bellefonte, PA, USA) with a vivinylbenzene/carboxen/polydimethylsiloxane fiber (divinylbenzene/Carboxen/polydimethylsiloxane (DVB/CAR/PDMS), 50/30 μm, coating 2 cm) (Supelco). The SPME fiber was conditioned by inserting it into a gas chromatography injection port at 220 °C for 1 h before extraction was conducted. Then, the SPME fiber was inserted into the vial containing the sample that was kept in a water bath at 60 °C for 30 min. The fiber was withdrawn into the needle and injected into the injection port of GC–MS at an injection temperature of 220 °C for 3 min. GC–MS analysis was conducted on a Varian CP-3800/Saturn 2000 (Varian, Wallnut Creek, CA, USA) equipped with a ZB-5MS capillary column (30 m × 0.25 mm inner diameter (i.d.) × 0.25 μm film thickness).

The initial oven temperature was kept at 50 °C, raised to 130 °C at a rate of 4 °C min^−1^, subsequently increased to 180 °C at 10 °C min^−1^, and finally increased to 280 °C at a rate of 2 °C min^−1^. Helium was used as a carrier gas at a flow rate of 1.0 mL min^−1^. The samples were manually injected in a split mode (split mode 1:10). Mass spectra were obtained in an electronic ionization (EI) mode of 70 eV with a scan range of *m*/*z* 35–550. After each extraction was completed, the SPME fiber was placed in the injection port of GC–MS at 220 °C for 15 min to ensure that volatiles were completely eluted to prepare for the subsequent extraction. Volatiles were identified by comparing the mass spectra of the compounds obtained experimentally, and the mass spectra were available in the NIST14 database. The retention index (RI) experimentally determined by Kovats was compared with the RI in NIST WebBook and literature data [43]. Quantification of identified compounds was calculated by comparing the peak area of individual compounds with the peak area of the standard used, *p*-methoxyphenol, with a concentration of 1 mg/mL.

### 3.9. Lipid Extraction

Lipid fraction was extracted in accordance with a method described previously [44] with minor modifications. Lipids from ground leaves were extracted with 2:1 chloroform–methanol (*v*/*v*) for 24 h and filtered. The extraction solvent was removed from the filtrate with a vacuum rotary evaporator at 65 °C. The obtained crude lipids were initially weighed and saponified with 10 mL of 0.5 M KOH/MeOH at 80 °C for 10 min, boiled with reflux for 5–10 min, and left to cool at 12 °C. The sample was then placed in a separation funnel. Hexane (10 mL) and water (10 mL) were sequentially added twice and vigorously mixed. For phase separation, 5 mL of saturated NaCl solution was added to the mixture and vigorously mixed. Fatty acids that partitioned to the polar phase were separated and removed from the phytosterol fraction.

### 3.10. Fatty-Acid Analysis

The fatty-acid fraction obtained in Section 3.9 was acidified with 1 M HCl and subsequently extracted with 10 mL of hexane. The organic portion was separated, and the extraction solvent was removed with a vacuum rotary evaporator. Methylation was performed by mixing with 4 mL of 14% BF_3_/MeOH (*v*/*v*) at 80 °C for 10 min. The formed fatty-acid methyl ester (FAME) was extracted with 2.5 mL of hexane, dried with MgSO_4_, and filtered with a cotton plug and silicate powder into a small container. The FAME profile was analyzed using a GC–MS (GCMS-QP 2020, Shimadzu, Kyoto, Japan). Separation was performed using a Zebron ZB-WAX capillary column (30 m × 0.25 mm i.d. × 0.25 μm film thickness) (Phenomenex, Torrance, CA, USA). Scanning was conducted at a mass range of 50–400 *m*/*z*, at an EI of 70 eV, and at a 5 scan s^−1^ mode. The carrier gas was helium with a flow rate of 1.0 mL min^−1^ at a split ratio of 1:10. The following temperature gradient programs were used: (a) 45 °C for 3 min, (b) 45 °C to 220 °C at a rate of 5 °C min^−1^, and (c) 220 °C to 250 °C at a rate of 10 °C min^−1^ and maintained at 250 °C for 2 min. The injector was held at 260 °C.

### 3.11. Phytosterol Analysis

The phytosterol fraction obtained in Section 3.11 was dried with MgSO_4_ and evaporated using a vacuum rotary evaporator to remove any solvent. The obtained residue was mixed with 0.2 mL of pyradine and 0.2 mL of BSTFA as a silylation agent in a tube. Then, approximately 1 mg of cholesterol was added as the internal standard. The mixture was heated in an incubator orbital shaker at 110 rpm and at 60 °C for 45 min. After derivitization was completed, the mixture was transferred to small capped tubes and analyzed with GC–MS. Phytosterols were identified by comparing their experimentally obtained mass spectra with the mass spectra available in literature, and by comparing the relative retention times of the standards. The concentrations of identified phytosterols were determined by comparing the peak area of each compound to the peak area of internal standard, cholesterol, with a concentration of 1 mg/mL.

### 3.12. Water Activity Analysis

The a_w_ of *S. crispus* leaves was measured with a water activity meter (Aqualab 4TE, Pullman, WA, USA) and analyzed at an average temperature of 24.9 ± 0.05 °C.

### 3.13. Color Measurement

The color of fresh and dried ground leaves was analyzed using a Minolta Chroma Meter CR-200 (Minolta Co. Ltd., Osaka, Japan), and data were expressed using L*, a*, and b* coordinates. Color measurements were repeated five times. Fragmented leaves were placed in different positions, and ground and dried leaves were mixed in each reading.

### 3.14. Statistical Analysis

Statistical analysis was performed using SPSS version 20 (IBM, Tulsa, OK, USA). Results were expressed as means ± standard deviation. Significant differences (*p* ≤ 0.05) between mean values were evaluated with Tukey’s test. Mathematical modeling was conducted with Table Curve 2D Windows v2.03 (Jandel Scientific Software, USA). Root-mean-square error and coefficient of determination (*R^2^*) were determined to evaluate the goodness of fit of mathematical models.

## 4. Conclusions

This study showed that CD and VMD show potential for producing dried forms of *S. crispus* with considerably high bioactivities. Suitable hot air temperature and microwave power are necessary to obtain high antioxidant activities, TPC, and total volatiles and phytosterols. The highest antioxidant activity was obtained for the samples dried with CD at 40 °C and VMD at 9 W/g, whereas the highest total phytosterols were achieved with VMD at 9 W/g. CD at 50 °C and 60 °C were the best options for volatile compounds and TPC, respectively. The drying kinetics of *S. crispus* for CD, VMD, and CPD-VMFD were best described by an exponential equation or the modified Page model. The specific energy consumption of VMD was the lowest among the methods because the total drying time was shortened throughout the heating duration of microwaves. The freeze-dried samples exhibited the lowest a_w_ and better color preservation.

## Figures and Tables

**Table 1:** Effect of drying methods and parameters on the antioxidant activity and total phenolic content (TPC) of *Strobilanthes crispus* leaves.

Drying Method	Antioxidant Activity (µM Trolox/100 g dw)		Total Phenolic Content (mg/100 g dw)
	ABTS	FRAP	
Fresh	6.10 ± 0.56 a	4.96 ± 0.31 a	1222.33 ± 75.87 a
FD	3.57 ± 0.37 b	4.48 ± 0.20 a,c	873.63 ± 13.86 b
VMD 6 W/g	3.97 ± 0.07 b,c	4.85 ± 0.07 a	898.88 ± 16.56 b
VMD 9 W/g	4.47 ± 0.21 c,e	5.61 ± 0.34 b	904.05 ± 41.90 b
VMD 12 W/g	3.46 ± 0.35 b	3.88 ± 0.09 c	830.37 ± 21.28 b,c
CPD-VMFD	2.65 ± 0.23 d	3.09 ± 0.32 d	734.45 ± 47.93 c,e
CD, 40 °C	5.08 ± 0.10 e	5.98 ± 0.21 b	1051.32 ± 25.36 d
CD, 50 °C	2.55 ± 0.00 d	2.99 ± 0.17 d	675.23 ± 12.02 e
CD, 60 °C	4.67 ± 0.17 c,e	5.67 ± 0.10 b	1086.71 ± 46.76 d

ABTS—2,2′-azinobis(3-ethylbenzthiazoline-6-sulfonic acid); FRAP—ferric-reducing antioxidant power; FD—freeze-drying; VMD—vacuum microwave drying; CPD—convective pre-drying; VMFD—vacuum microwave finish drying; mean values with different letters within the same column were significantly different (*p* < 0.05).

**Table 2:** Concentration of volatile compounds influenced by drying methods.

Compound	Peak	RT	Retention Indexes		Fresh	FD	CPD-VMFD	CD			VMD		
			Exp.	Lit.				40 °C	50 °C	60 °C	6 W/g	9 W/g	12 W/g
					Concentration (mg 100 g^−1^ dry basis (db))								
**Isopentyl alcohol**	1	2.861	736	736	3.97	0.17	0.00	nd	0.02	0.02	0.11	0.03	0.00
**Pyridine**	2	3.015	743	746	12.45	0.05	0.02	0.00	0.05	0.02	0.06	0.01	0.05
**2-Penten-1-ol, (*Z*)-**	3	3.322	768	769	0.63	0.04	0.10	0.02	0.09	0.13	0.23	0.04	0.07
**2-Hexen-1-ol, (*E*)-**	4	3.754	854	857	10.18	0.58	1.65	0.93	7.21	2.85	1.03	1.77	1.07
**2-Hexenal, (*E*)-**	5	4.763	864	854	51.18	0.93	1.13	0.85	2.00	0.74	0.88	0.36	0.15
**(*Z*)-Hex-3-en-1-ol**	6	4.820	867	857	15.31	1.29	0.79	0.63	1.50	0.87	0.36	0.17	0.05
**2-Hexen-1-ol, (*Z*)-**	7	5.098	878	868	84.11	2.13	0.51	0.40	0.61	0.75	0.53	0.23	0.18
**2,4-Hexadienal**	8	6.089	910	911	4.22	0.06	0.05	0.08	0.15	0.10	0.07	0.11	0.03
**Unknown**	9	6.672	930	-	0.45	0.14	0.01	nd	0.00	0.01	0.03	0.11	0.08
**Benzaldehyde**	10	7.497	969	962	27.61	0.40	0.21	0.32	0.57	0.66	0.33	0.24	0.06
**1-Octen-3-ol**	11	8.039	977	980	36.10	1.30	0.68	0.75	0.40	1.57	0.62	0.22	0.15
**3-Octanone**	12	8.248	985	986	13.23	1.14	0.46	0.27	0.54	0.83	0.53	0.31	0.20
**3-Octanol**	13	8.527	995	994	8.97	0.48	0.45	0.22	0.32	0.56	0.39	0.24	0.09
**2,4-Heptadienal**	14	8.956	1009	1011	0.64	0.02	0.10	0.01	0.12	0.06	0.03	0.08	0.02
**1-Cyclohexene-4-carboxaldehyde, 1-methyl**	15	9.234	1024	1017	1.78	0.02	0.03	0.01	0.00	0.01	0.01	0.08	0.10
***p*-Cymene**	16	9.470	1024	1025	2.03	0.55	0.05	0.05	0.16	0.05	0.14	0.00	0.00
**Limonene**	17	9.625	1029	1030	3.05	0.04	0.27	0.03	0.05	0.04	0.09	0.01	0.02
**Eucalyptol**	18	9.707	1032	1032	0.37	0.00	0.02	0.03	0.04	0.01	0.01	0.11	nd
**Benzyl alcohol**	19	9.777	1034	1036	8.05	0.17	0.11	0.15	0.21	0.28	0.19	0.12	0.03
**3-Octen-2-one**	20	9.849	1037	1040	0.12	0.03	0.11	0.11	0.26	0.12	0.13	0.18	0.04
**Benzeneacetaldehyde**	21	10.001	1042	1045	3.02	0.36	0.21	0.11	0.13	0.29	0.37	0.18	0.06
**2-Octenal**	22	10.512	1056	1060	1.02	0.03	0.06	nd	0.24	0.11	0.06	0.05	0.04
**Acetophenone**	23	10.833	1064	1065	1.43	0.02	0.01	0.01	0.00	0.01	0.01	0.00	0.01
**3,5-Octadien-2-one**	24	10.956	1069	1073	3.09	0.25	0.68	0.53	1.26	1.25	1.03	0.77	0.42
***cis*-Linalool oxide**	25	11.053	1072	1074	0.60	0.03	0.03	0.01	0.01	0.10	0.08	0.04	0.01
**β-Phorone**	26	11.221	1079	-	1.01	0.01	0.04	0	0.02	0.06	0.04	0.18	0.01
**Linalool**	27	11.958	1100	1100	34.91	1.28	0.69	0.88	0.39	0.93	0.50	0.23	0.09
**2-Nonen-1-ol**	28	12.097	1103	1105	5.49	0.17	0.17	0.19	0.25	0.37	0.31	0.56	0.07
**3-Octen-2-ol**	29	12.262	1108	-	1.95	0.18	0.79	0.31	0.44	1.40	0.88	0.47	0.31
**Phenylethyl Alcohol**	30	12.415	1114	1116	6.51	0.29	0.18	0.23	0.00	0.28	0.30	0.12	0.05
**Isopulegol**	31	13.505	1147	1146	3.41	0.04	0.09	0.11	0.15	0.08	0.13	0.00	0.07
**Methyl salicylate**	32	15.224	1196	1192	4.31	0.66	0.27	0.17	0.03	0.27	0.04	0.36	0.22
**Ethyl salicylate**	33	17.784	1271	1270	9.12	0.07	0.06	0.08	0.09	0.05	0.13	0.09	0.02
**Pentanoic acid,** **heptyl ester**	34	21.016	1378	1376	0.89	0.04	0.08	0.28	0.08	0.11	0.14	0.36	0.05
**TOTAL**					361.23	12.97	10.10	7.76	17.40	15.00	9.80	7.84	3.80

RT—retention time; RI—retention index; Exp—experimental; Lit—literature; FD—freeze-drying; CD—convective drying; VMD—vacuum microwave drying; CPD—convective pre-drying; VMFD—vacuum microwave finish drying; nd—not detected.

**Table 3:** Concentration of phytosterols influenced by drying methods.

Compound	Retention Time		Fresh	FD	CPD-VMFD	CD 40 °C	CD 50 °C	CD 60 °C	VMD 6 W/g	VMD 9 W/g	VMD 12 W/g
	Exp.	Lit.	Concentration (mg 100 g^−1^ db)								
**α-tocopherol**	25.950	25.950	210.40	13.19	17.57	10.72	20.02	10.94	11.04	19.97	12.88
**Desmosterol**	26.630	26.630	254.54	30.17	37.34	25.15	27.62	30.03	27.29	23.26	28.78
**Lanosterol**	26.875	26.880	148.60	20.60	22.93	18.49	23.54	22.34	23.81	20.07	22.39
**Campesterol**	27.575	27.580	791.57	68.88	86.53	61.79	60.06	81.48	56.23	66.77	59.21
**Stigmasterol**	28.035	28.150	1207.96	141.55	140.74	119.26	135.32	144.80	135.76	147.60	142.90
**β-sitosterol**	28.955	28.980	1476.47	157.16	153.65	201.10	228.01	194.95	186.08	229.18	207.87
**β-amyrin**	29.225	29.190	230.26	26.64	26.97	32.32	35.59	37.17	33.38	30.38	31.80
**Cycloartenol**	30.055	30.050	34.58	1.47	3.25	2.94	1.39	2.41	1.30	3.19	1.92
**Betulin**	31.225	31.170	61.06	2.41	1.93	1.81	3.15	2.87	2.09	2.06	1.10
**TOTAL**			4415.44	462.07	490.91	473.59	534.71	527.00	476.99	542.48	508.84

Exp—experimental, Lit—literature, FD—freeze-drying, CD—convective drying, VMD—vacuum microwave drying, CPD—convective pre-drying, VMFD—vacuum microwave finish drying.

**Table 4:** Profile of fatty acids in *S. crispus*.

Compound	Retention Time	Total Area %
Capric acid	18.750	0.07
Lauric acid	23.500	0.41
Tridecanoic acid	25.665	0.20
Myristic acid	27.810	1.74
Pentadecanoic acid	29.830	0.18
Palmitic acid	31.745	10.48
Palmitoleic acid	32.130	1.93
Hexadecenoic acid, methyl ester, (11Z)-	32.655	1.44
Heptadecanoic acid	33.590	0.21
*cis*-10-Heptadecenoic acid	33.910	0.15
Stearic acid	35.365	6.07
Oleic acid	35.640	3.61
Elaidic acid	35.770	0.30
Linoleic acid	36.380	12.49
α-Linolenic acid	37.415	58.00
Arachidic acid	38.675	0.40
Behenic acid	41.170	1.20
Erucic acid	41.470	0.12
*cis*-4,7,10,13,16,19-Docosahexaenoic acid	42.665	1.00

**Table 5:** Water activity of dried *S. crispus* dehydrated using different methods.

Drying Method	Water Activity, a_w_
Fresh	0.9879 ± 0.000 a
Freeze-drying	0.0245 ± 0.000 b
CD 40 °C	0.1577 ± 0.010 c
CD 50 °C	0.1071 ± 0.004 d,f
CD 60 °C	0.0968 ± 0.004 d,e
VMD 6 Wg^−1^	0.1539 ± 0.002 c
VMD 9 Wg^−1^	0.0821 ± 0.005 e
VMD 12 Wg^−1^	0.1239 ± 0.003 f,g
CPD-VMFD, 50 °C, 9 Wg^−1^	0.1272 ± 0.004 g

CD—convective drying, VMD—vacuum microwave drying, CPD—convective pre-drying, VMFD—vacuum microwave finish drying. Values with the same letter in the same column were not significantly different (*p* < 0.05), according to Tukey’s test.

**Table 6:** Color parameters L*, a* and b* of *S. crispus* affected by drying methods.

Drying Conditions	Color Parameters		
	L*	a*	b*
Fresh	35.54 ± 0.504 a	−2.98 ± 0.309 a,d	6.42 ± 0.414 a
Freeze drying	43.93 ± 0.380 b	−3.39 ± 0.064 b	10.67 ± 0.172 b
CD 40 °C	43.20 ± 0.131 c	−3.32 ± 0.055 b,e	9.68 ± 0.163 c
CD 50 °C	41.20 ± 0.365 d	−2.69 ± 0.068 c	8.35 ± 0.053 d
CD 60 °C	41.70 ± 0.078 d,e	−2.75 ± 0.058 c,d	8.84 ± 0.126 e
VMD 6 W/g	43.24 ± 0.128 c	−3.37 ± 0.110 b	10.13 ± 0.074 f,g
VMD 9 W/g	43.30 ± 0.196 c	−3.39 ± 0.088 b	9.84 ± 0.133 c,f
VMD 12 W/g	42.96 ± 0.092 c	−3.18 ± 0.055 a,b,e	10.25 ± 0.090 g
CPD-VMFD	42.01 ± 0.190 e	−3.10 ± 0.080 a,e	9.24 ± 0.093 h

CD—convective drying; VMD—vacuum microwave drying; CPD—convective pre-drying; VMFD—vacuum microwave finish drying; values with the same letter within a column were not significantly different (*p* < 0.05), according to Tukey’s test.

**Table 7:** Mathematical models applied to drying curves of *Strobilanthes crispus*.

Model Name	Model Equation
Lewis	MR=exp(−k·t)
Modified Page	$MR=a\cdot \exp\left(-k\cdot t^{n}\right)$
Henderson and Pabis	MR=a·exp(−k·t)

*MR*—moisture ratio; *a*—coefficient of the equation; *k*—drying constant (min^−1^); *n*—exponent; *t*—time (min).
